# Gait Estimation from Anatomical Foot Parameters Measured by a Foot Feature Measurement System using a Deep Neural Network Model

**DOI:** 10.1038/s41598-018-28222-2

**Published:** 2018-06-29

**Authors:** Kyung-Ryoul Mun, Gyuwon Song, Sungkuk Chun, Jinwook Kim

**Affiliations:** 1 Imaging Media Research Center, Korean Institute of Science and Technology, Seoul, Republic of Korea; 2 Spatial Optical Information Research Center, Korea Photonics Technology Institute, Gwangju, Republic of Korea

## Abstract

An accurate and credible measurement of human gait is essential in multiple areas of medical science and rehabilitation. Yet, the methods currently available are not only arduous but also costly. Researchers who investigated the relationship between foot and gait parameters have found that the two parameters are closely interrelated and suggested that measuring foot characteristics can be an alternative to the strenuous quantification currently in use. This study aims to verify the potential of foot characteristics in predicting the actual gait temporo-spatial parameters and to develop a deep neural network (DNN) model that can estimate and quantify the gait temporo-spatial parameters from foot characteristics. The foot features in sitting, standing, and one-leg standing conditions of 42 subjects were used as the input data and gait temporo-spatial parameters at fast, normal, and slow speed were set as the output of the DNN regressor. With the prediction accuracy of 95% or higher, the feasibility of the developed model was verified. This study might be the first in attempting experimental verification of the foot features serving as predictors of individual gait. The DNN regressor will help researchers improve the data pool with less labor and expense when some limitations get properly overcome.

## Introduction

Gait is the most basic form of human locomotion migrating the body’s center of mass (CoM) in various directions and it contains much personal information such as movement patterns, pathological symptoms, and movement intentions^1–3^. Therefore, accurate and reliable gait quantification is indispensable in making proper and timely clinical intervention. Gait quantification not only provides substantial clues in diagnosing and monitoring muscular skeletal diseases and neurological disorders but also helps evaluate the life quality of the suffering^4,5^.

Measuring human gait extensively rely on temporo-spatial characteristics of an individual such as the time and length of stride and step, stance time, swing time, single-limb support (SLS) time, double-limb support (DLS) time, and gait velocity^1,6–8^. Those characteristics can be measured by optical motion capture system^1,8^, or floor sensors^9,10^. Since they are special devices, a huge cost is unavoidable. Let alone the cost, this kind of measuring requires strict laboratory environment that hampers natural gait of subjects and can be easily disturbed.

To address the challenge, a wearable sensor like inertial-measurement unit (IMU) was developed and now it is widely in use. Although it requires neither a huge budget nor sophisticated experimental settings, it is accurate and reliable^11,12^. However, some challenges still remain. A single IMU has not yet fully overcome a drift phenomenon despite its significant reduction due to several supplementary techniques, thus cannot provide accurate position information while subjects are performing continuous motion tasks. Commercialized IMU-based motion capture systems combining several IMU sensors can be a good alternative option for kinematic measurements. However, some issues such as constraining human kinematics, sensor fusion technique, magnetic disturbance, making a relation between the sensors and anatomical body segment frames through calibration, and detecting foot contact and off time make the measurement process complicate^2,13–15^.

In the interest of finding a better way of measuring human gait, many attempts have been made. Some of them turned attention to the prediction potential of artificial neural networks. Ardestani and his colleagues developed a generic wavelet neural network (WNN) model that predicts human joint moments and verified its accuracy by comparing its prediction with that of feed-forward artificial neural network (FFANN) model^16^. Based on the accuracy rate that has less than 10 percent of normalized root mean square error (RMSE), Ardestani argued that his WNN model can predict joint moments more conveniently yet accurately compared to the conventional multi-body dynamic models. Another study conducted by Yun *et al*. adopted a statistical and stochastic approach and used anthropometric data of a human body and estimated joint kinematics while walking^17^. This novel approach lowered the estimation cost greatly by using subject-specific body anthropometric parameters. Some other researchers like Hannink *et al*. used convolutional neural network (CNN) model and successfully predicted the biomechanical stride parameters with comparable accuracy^2,18^. A recurrent neural network (RNN) model was also studied and the results said it successfully detects the movement intentions of the five major movements which are closely related to daily tasks^3^. All these studies support the prediction potential of neural networks and their compatible accuracy. These remarkable achievements endorse an investigation of yet another neural network, a deep neural network (DNN), in measuring human movement. The rise of artificial intelligence technology can also help find ways that are more efficient in quantifying and classifying human movements.

This newly emerged use of neural networks in quantifying human movements inevitably poses a question of input data. Accordingly, many studies investigating the use of certain features as input data followed. One of the candidates is a foot since its arch structure contains much information on how individuals walk, and the foot is highly associated with the whole body dynamics including plantar load distribution^19–21^. Chang *et al*. found that the height of foot arch changes weight distribution^22^. The study compared the weight distribution of people with low foot arch with that of people with normal arch then concluded that people with low foot arch tends to shift their body more on the medial side while walking. Another study conducted by Sung *et al*. found that people with low foot arch had increased external hip rotation and decreased forefoot supination angle^23^. By showing the kinematic differences caused by foot arch types, these studies imply the causal connection between the two. More specifically, a study conducted by Mun *et al*. has proven the correlation between the foot feature and the gait temporo-spatial parameters^24,25^. When foot feature parameters measured by a newly developed foot feature measurement system (FFMS) and gait temporo-spatial parameters collected from a motion capture system were investigated, it was found that medial-longitudinal arch (MLA) and lateral-longitudinal arch (LLA) can move independently despite their physical proximity. As for their correlations, it was found that the MLA characteristics are correlated with the gait temporal parameter while the LLA characteristics are correlated with gait spatial parameters. This correlation found by multiple studies advocates the use of foot features as input data in predicting individual gait patterns.

This study aimed to develop a neural network model that predicts human gait and verify its accuracy. A deep neural network based regressor using foot characteristics as input was built and it estimated and quantified the gait temporo-spatial parameters. The estimated gait temporo-spatial parameters were compared with the actual values. The study also looked for the most applicable and reliable input variable set among the studied variable sets. Measuring foot characteristics is much simpler and cheaper than collecting gait-related parameters. When the accuracy of the suggested model is verified, it can serve as a good alternative to the sophisticated measurement currently available. This new subject-specific gait estimation approach will dramatically reduce the cost and effort that accompany the quantification human gait.

## Methods

### Participants and Experimental Protocols

Based on the assumption that the foot arch characteristics of regular people and athletes differ distinctively^26^, a total of 42 subjects, 17 regular subjects (age: 29.41 ± 5.08, height: 174.94 ± 4.87, and weight: 73.35 ± 7.98) and 25 semi-professional athletes (age: 52.92 ± 9.60, height: 171.96 ± 4.8, and weight: 69.76 ± 5.36), were recruited. The recruited semi-athlete subjects run a triathlon or marathon at least once in three months. Subjects who had any history of musculoskeletal injuries, neurological disorder, and age-related health issues were excluded. The experimental protocol was assessed and approved by the Intuitional Review Board of Korea Institute of Science and Technology. All methods were performed in accordance with the relevant guidelines and regulations. The informed consent was obtained from all participants and no violation of human right was reported.

**Figure 1:**
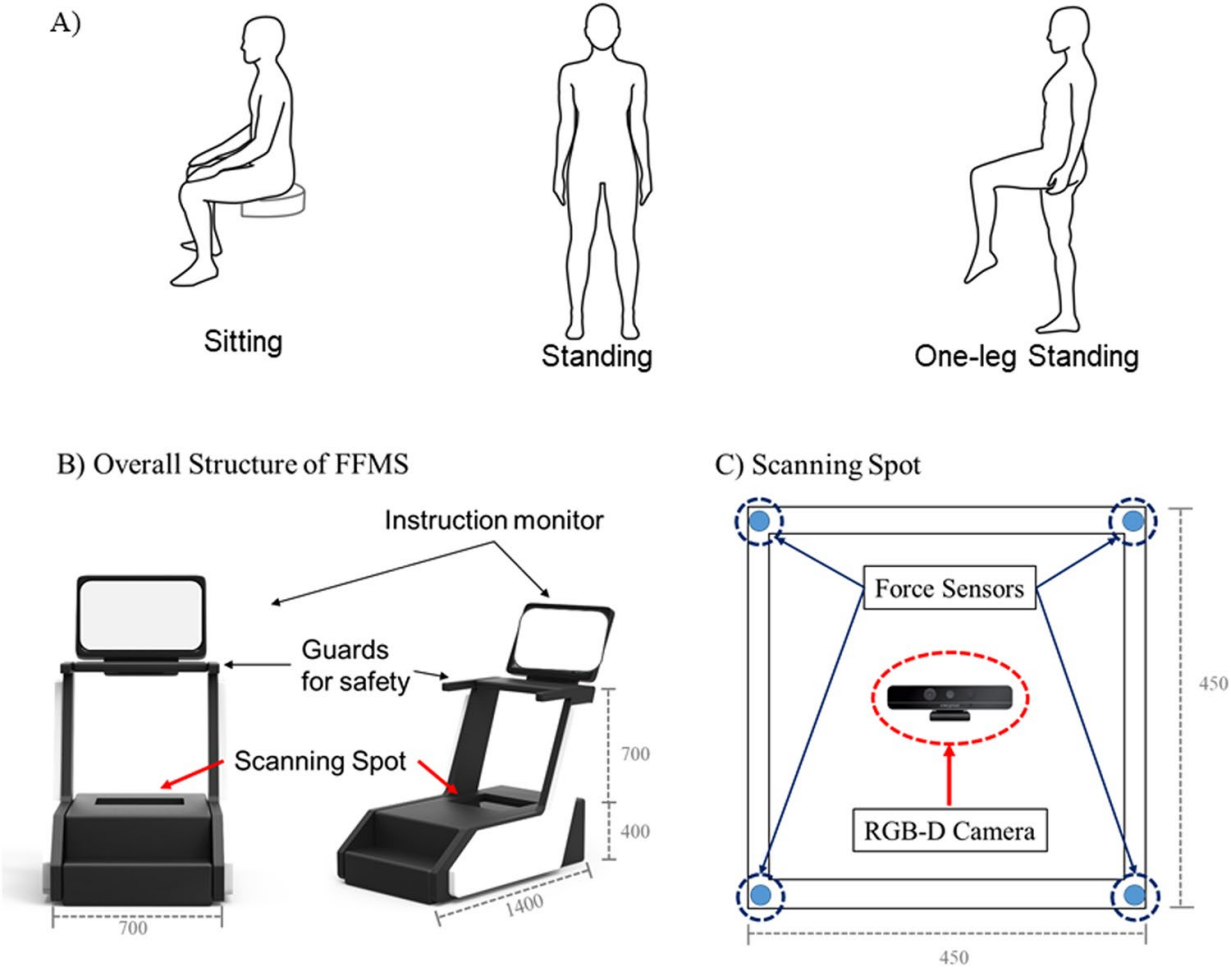
(**A**) Experimental protocols of foot feature measurement session under sitting, standing, and one-leg-standing conditions. (**B**) Overall scheme of the foot feature measurement system (FFMS). FFMS has a standing type structure and a monitor installed on the front side. On both sides there are guard handles and there is the scanning spot on the bottom. (**C**) The scanning spot comprises four uniaxial force sensors at each corner of a transparent panel to measure center of pressure of the body and a single RGB-depth camera to collect foot structural information.

The protocols had two sessions that are foot feature measurement session and gait feature measurement session. The foot feature measurement session comprised three movement conditions: sitting, standing, and one-leg-standing (OLS) (Fig. 1A). The gait feature measurement session had three speed conditions: fast, normal, and slow. During the foot feature measurement session, all subjects were instructed to sit and maintain a sedentary posture with their ankle and knee joint angle at 90° and then steadily stand up and maintain the standing posture for 5 seconds on the system developed. Followed by these, the subjects were asked to stand on one leg keeping their body balance as stable as possible for 10 seconds (Fig. 1A). The foot feature measurement session was performed on a newly developed foot measurement system which can provide foot shape as well as the ground reaction force during movements. The details of the system are shown in Fig. 1. The foot features were collected when the ground reaction force was the highest and lowest, then the averaged values were used for the analysis. During the gait feature measurement session, the subjects were asked to walk on a 30-meter long straight path three times: once at their preferred speed, and 15 to 25% slower and faster than their casual speed. Ten strides in the middle of these were used for the analysis. For this study, a total of 61 samples were gathered. For the regular subjects, both foot and gait features were collected from one dominant limb and those of the semi-athletes were collected from both limbs.

### A Foot Feature Measurement System (FFMS) and Foot Data Analysis

**Figure 2:**
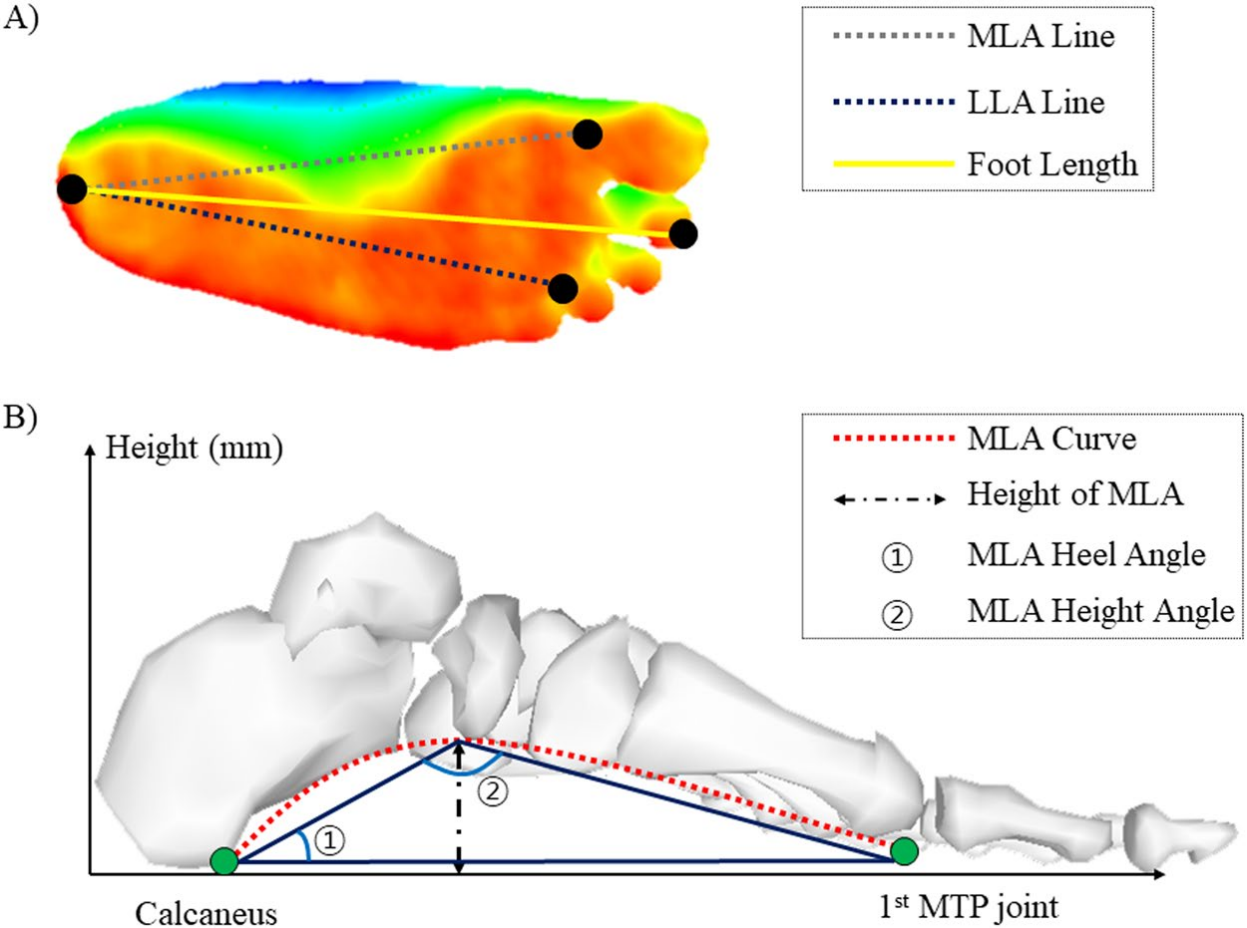
(**A**) A distance between a center of heel and center of second toe is defined as foot length, while a center of heel to 1^st^ MTP bone and to 4^th^ MTP bone are defined as MLA and LLA line, respectively. (**B**) From the MLA and LLA line, foot arch parameters such as curves, height, heel and height angles of MLA and LLA were calculated.

The overall scheme of the FFMS (1400 mm (length) × 700 mm (width) × 1100 mm (height)) is presented in Fig. 1B. The system has a standing type structure with a monitor that displays user instructions. There are guard handles on both sides and the scanning spot on the bottom. The FFMS measures the foot structure of a subject while subjects are performing various motion tasks on the scanning spot and from the gathered structural data foot feature parameters such as foot length, foot width, MLA and LLA curves get extracted. The scanning spot is equipped with four uniaxial force sensors (Phidgets Inc., Calgary, Canada) at each corner of a colorless and transparent acrylic panel (450 mm (length) × 450 mm (width) × 400 mm (height)) to measure the center of pressure (CoP) of a body. Underneath the panel, there is a single RGB-depth (RGBD) camera (Realsense F200, Intel, Santa Clara, USA) for collecting the structural information of a foot (Fig. 1B). The RGBD camera captures 3D geometric and color data of a plantar surface with 60 frames per second in point cloud data format. The foot length was defined as the distance from a center of heel to center of the second toe. The distance from a center of heel to 1^st^ metatarsophalangeal (MTP) bone was defined as MLA line while the distance from a center of heel to 4th MTP bone was defined as LLA line (Fig. 2A). Then by projecting MLA and LLA lines onto the plantar surface, the MLA and LLA curves were computed. From these curves, the parameters such as foot length, height and curve area of the MLA and LLA and arch angles were calculated (Fig. 2B). The accuracy and feasibility of FFMS were validated from our previous study^25,27^.

### A Gait Measurement System and Gait Temporo-spatial Parameters

**Figure 3:**
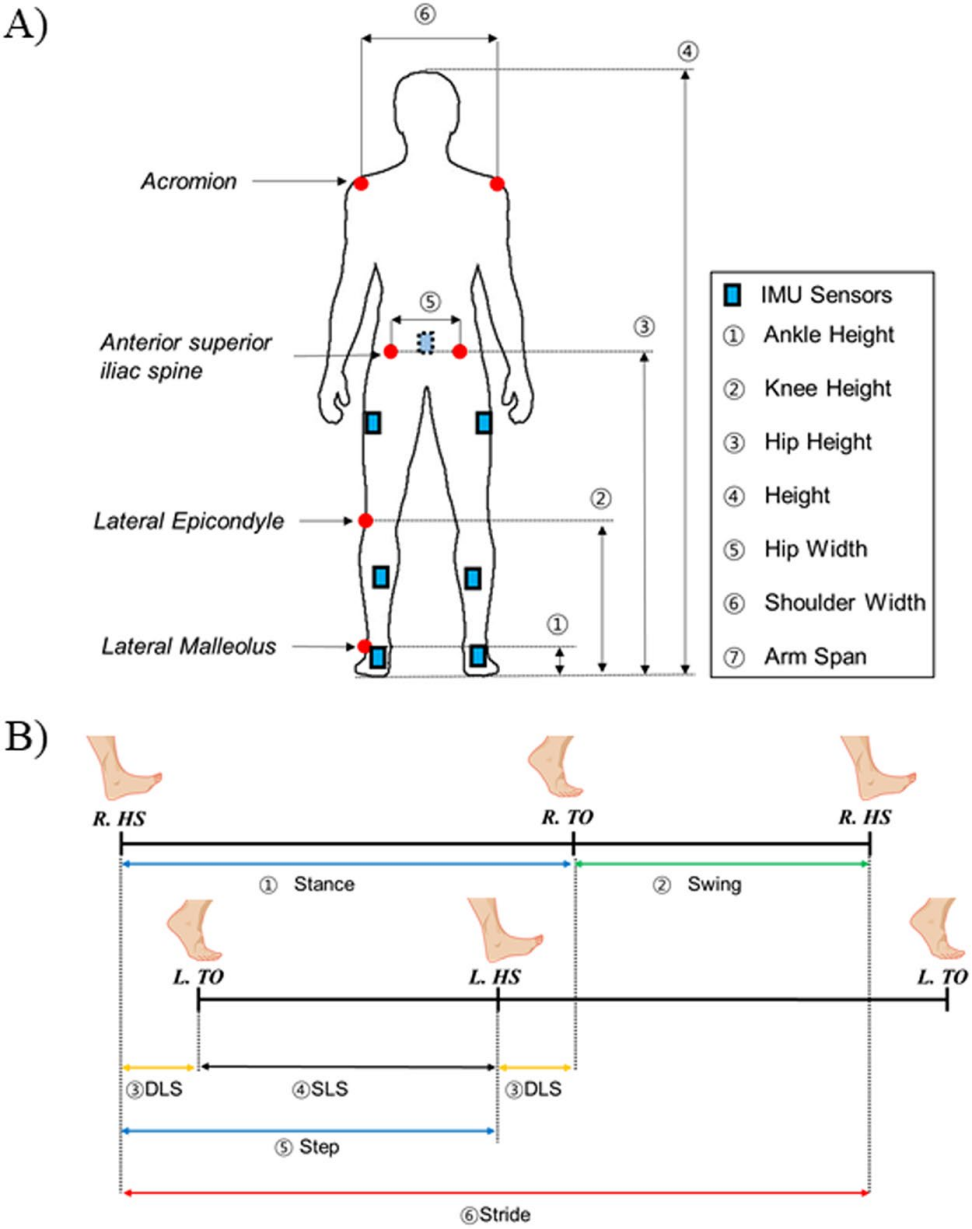
(**A**) Experimental setting for the gait experiment with IMU sensors mounted on a subject, and the subject**’**s anthropometric data such as ankle height, knee height, hip height, height, hip width, shoulder width, and arm span collected prior to the gait experiment. (**B**) Definitions of gait temporo-spatial parameters such as stance, swing, step, stride, single-limb support and double-limb support.

Before measuring the gait of the subjects, the anthropometric data of each subject such as ankle height, knee height, hip height, body height, hip width, shoulder width, and arm span were measured (Fig. 3A). A commercialized motion-capture system (Xsens MVN, Enschede, Netherland) equipped with IMU sensors was used to collect gait-related information (Fig. 3A)^11,12^. The angular velocity of left and right shanks was used to detect heel-strike (HS) and toe-off (TO) time of both lower limbs^28,29^. From these detected HS and TO, the gait temporo-spatial parameters were calculated (Fig. 3B). The phase from HS to TO was defined as a stance and TO to consecutive HS was defined as a swing phase. Other definitions of double-limb support (DLS), single-limb support (SLS), step, stride, and gait velocity can be found in Fig. 3B^4^.

### Deep Neural Network based Regressor and Training and Evaluation scheme

**Figure 4:**
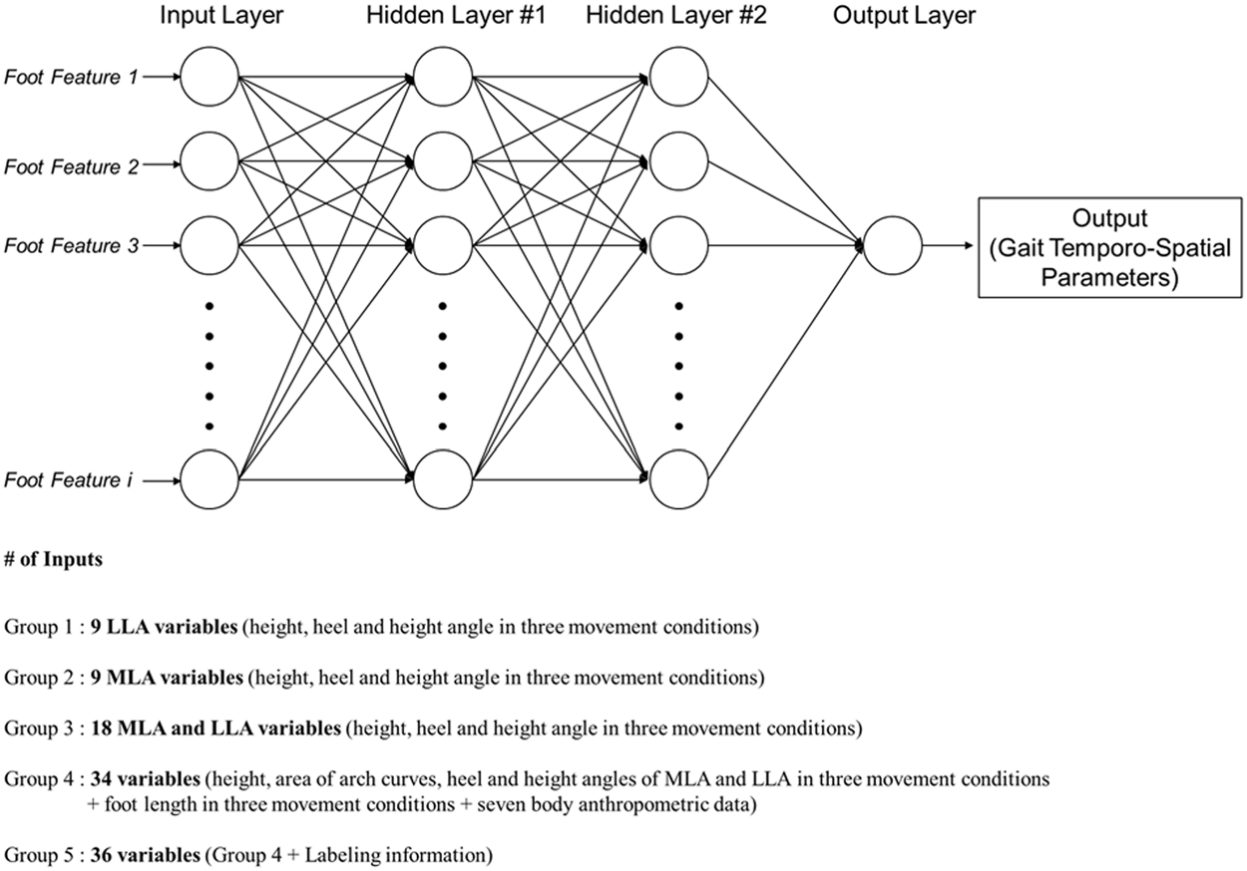
The structure of DNN regressor is determined by the grid search algorithm. It has 2 hidden layers having ‘Adam’ optimizer, ‘ReLU’ activation function with 0.1 learning rate and 1000 epochs for training iteration. The number of neurons is the same with the input features.
(D) Train deep neural networks: The training data set is fed into the established neural networks, and the networks calculate Mean-Squared-Error (MSE) and standard deviation (SD) between an actual value and an estimated value for each combination of weights and biases. When the training finishes, the network chooses an optimal model which has a minimum MSE.
(E) Validate the optimal model: Using a new set of input data, the optimal model estimates gait parameters and compares the values with the corresponding actual values.

Using the DNN model which has been showing remarkable performances in various fields including gait kinematics and kinetics^30–32^, a DNN based regressor model was developed using Python software to estimate the gait temporo-spatial parameters. Input variables for the neural network were foot feature parameters of each subject which were measured by the FFMS under sitting, standing, and OLS conditions. The outputs were the average of the gait temporo-spatial parameters for each speed condition. The regressor estimated the gait parameters following five steps.

(A) Scale an input data set: Since the measurement units for foot and those of body features differ, all the input variables get scaled. A standard scaling approach which sets a mean of the inputs to 0 and a variance of the inputs to 1 is used.
(B) Shuffle and divide data: All acquired data get shuffled then are divided into learning set and test set as 7:3 ratios. At each learning stage, Python Deep Learning Library in Keras cross-validated the dataset to avoid overfitting.
(C) Build deep neural networks: Network architecture such as the number of layers, the number of neurons, learning rate, activation function, and so on, get configured (Fig. 4).

Using the regressor, several experiments in various settings were conducted. The input variables were classified into five groups to investigate which dataset is the most proper and optimized input set among the anthropometric data on foot and body. Whereas only the LLA related parameters for group 1 and MLA related parameters for group 2 were selected, the combination of MLA and LLA were selected for group 3. All foot parameters and body anthropometric parameters were selected for group 4, while all foot parameters, body anthropometric data, and labeling information that tells whether a subject is the regular or semi-athletes were selected for group 5. For group 1 and 2, the total of nine variables including the height, heel angle, and height angle of LLA and MLA in sitting, standing, and OLS conditions were selected as the inputs of the regression model. Eighteen variables from MLA and LLA characteristics were selected for group 3. For group 4, a total of 34 variables including foot length, height and area of the arch curves, heel and height angle of MLA and LLA in all three movement conditions as well as seven body anthropometric data were used. All the variables selected for group 4 and labeling information were used for group 5.

The prediction outputs of the regressor were 27 gait temporo-spatial parameters such as stride and step time, SLS and DLS time, stance and swing time, stride and step length, and gait velocity at fast, normal, and slow walking speed.

To find an optimal architecture of DNN, we adopted grid search algorithm that can perform an exhaustive search and hyper-parameter optimization. During the grid search, the number of hidden layers was selected at a range from 1 to 5, and the number of neurons was searched at a range between 1 and 5 times the number of input features. Seven candidates such as ‘SGD’, ‘RMSprop’, ‘Adagrad’, ‘Adadelta’, ‘Adam’, ‘Adamax’, ‘Nadam’ were considered for the optimizers, while eight candidates such as ‘softmax’, ‘softplus’, ‘softsign’, ‘ReLU’, ‘tanh’, ‘sigmoid’, ‘hard_sigmoid’, ‘linear’ were examined for the activation functions. The learning rate and epochs were determined by our empirical judgment. For the detailed information of each hyper-parameter, refer to Keras library document (https://keras.io/). After doing some grid search with a few randomly selected samples, we set the architecture of 2 hidden layers having ‘Adam’ optimizer, ‘ReLU’ activation function with 0.1 learning rate and 1000 epochs for training iteration considering a trade-off between accuracy and cost. The number of neurons was the same as the number of input feature (Fig. 4). The total of 61 samples was randomly classified into the learning set (70%) and the test set (30%). The MSE and SD were used in evaluating the performance of the regressor model.

## Results

**Figure 5:**
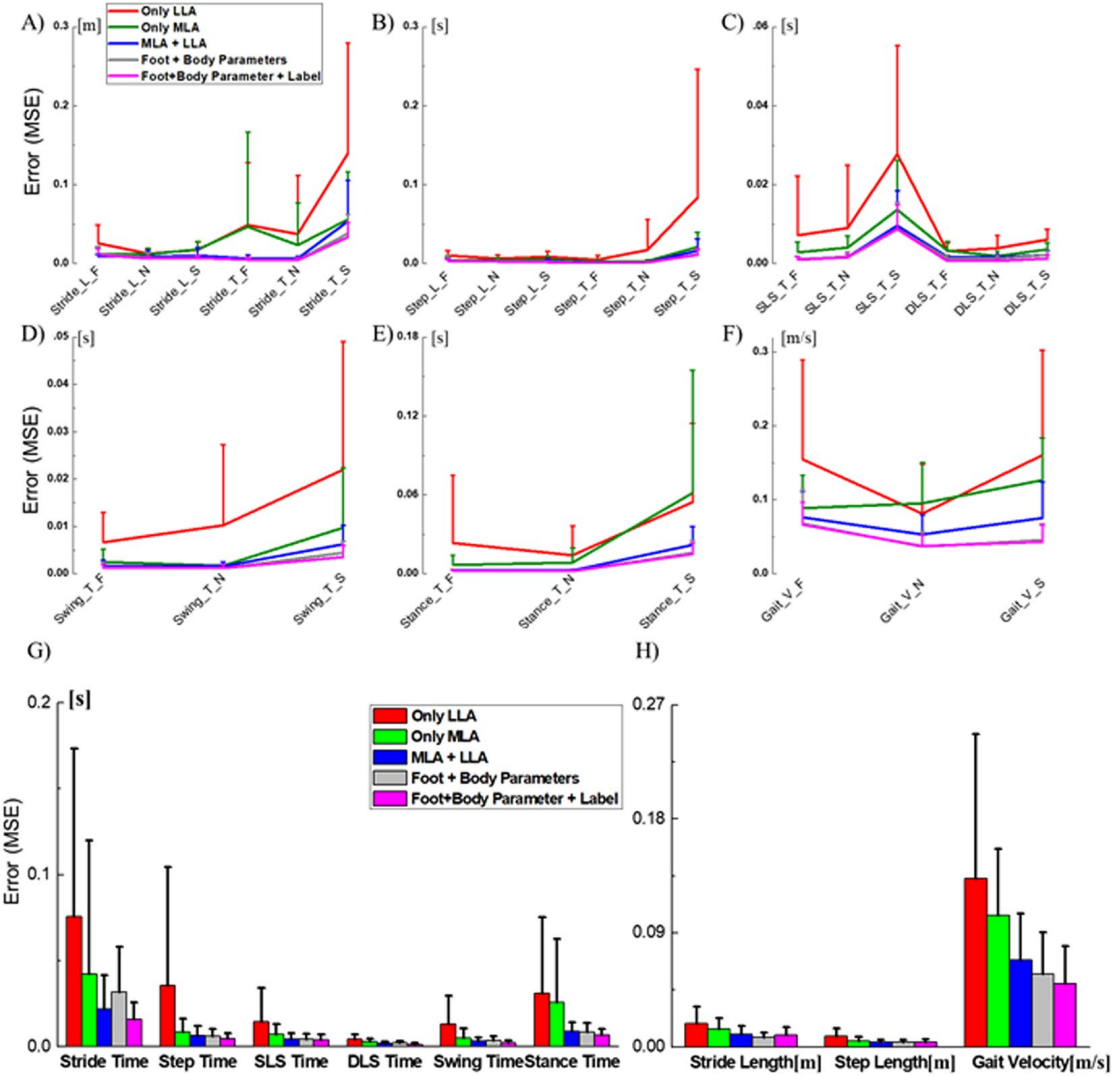
(**A** to **F**) Mean square errors and their standard deviation of the gait temporo-spatial parameters at fast, normal, and slow walking speed. (**G** and **H**) The mean square errors and their standard deviation of each gait temporal and spatial parameters averaged at various gait speeds on the unseen test data (out-of-sample) of the DNN based regressor model.

Figure 5 shows the MSEs of the gait temporo-spatial parameters at each speed condition. The MSEs and SDs of gait temporal and spatial parameters averaged were shown in Fig. 5G,H) and Table 1. The averaged prediction accuracies of the gait temporo-spatial parameters were shown in Table 2. When the numbers of input on the regressor increased, the prediction errors remarkably decreased.

**Table 1:** The mean square errors and their standard deviations averaged at various gait speeds.

	Group 1		Group 2		Group 3		Group 4		Group 5	
	Mean	Std	Mean	Std	Mean	Std	Mean	Std	Mean	Std
Stride Length [m]	0.019	0.013	0.014	0.009	0.010	0.007	0.007	0.004	0.009	0.006
Step Length [m]	0.008	0.006	0.005	0.003	0.004	0.002	0.004	0.002	0.004	0.002
Gait Velocity [m/s]	0.133	0.114	0.104	0.053	0.068	0.037	0.058	0.033	0.050	0.029
Stride Time [s]	0.076	0.010	0.042	0.078	0.022	0.020	0.032	0.026	0.016	0.010
Step Time [s]	0.036	0.069	0.009	0.008	0.006	0.006	0.006	0.004	0.005	0.003
SLS Time [s]	0.015	0.019	0.007	0.006	0.004	0.004	0.004	0.003	0.004	0.003
DLS Time [s]	0.004	0.003	0.003	0.002	0.002	0.001	0.002	0.001	0.001	0.001
Swing Time [s]	0.013	0.017	0.005	0.006	0.003	0.002	0.004	0.002	0.002	0.001
Stance Time [s]	0.031	0.044	0.026	0.037	0.009	0.005	0.009	0.005	0.007	0.004

**Table 2:** The prediction accuracies averaged at various gait speeds (%).

	Group 1	Group 2	Group 3	Group 4	Group 5
Stride Length	97.80	98.31	98.86	98.92	99.01
Step Length	98.00	98.88	99.19	99.18	99.39
Gait Velocity	85.35	88.35	92.51	94.69	94.82
Stride Time	93.44	96.16	98.14	98.66	98.79
Step Time	94.18	98.55	98.95	99.24	99.24
SLS Time	97.11	98.66	99.20	99.24	99.27
DLS Time	94.68	96.21	97.72	97.93	98.95
Swing Time	97.42	99.09	99.38	99.53	99.61
Stance Time	95.18	96.22	98.69	98.99	99.04

For the gait temporal parameter estimation, the MSEs and SDs of the stride and step time, SLS and DLS time, swing and stance time of group 1 were 0.076 ± 0.098, 0.036 ± 0.069, 0.015 ± 0.019, 0.004 ± 0.003, 0.013 ± 0.017, and 0.031 ± 0.044, respectively. Those of group 3 were 0.022 ± 0.020, 0.006 ± 0.006, 0.004 ± 0.004, 0.002 ± 0.001, 0.003 ± 0.002, and 0.009 ± 0.005 while those of group 5 were 0.016 ± 0.010, 0.005 ± 0.003, 0.004 ± 0.003, 0.001 ± 0.001, 0.002 ± 0.001, and 0.007 ± 0.004 (Table 1). The MSEs and SDs were the highest in group 1 and the lowest in group 5. The MSEs of group 3, 4, and 5 were significantly lower than those of group 1 and 2. The averaged accuracies of each temporal outputs were 93.44, 94.18, 97.12, 94.68, 97.42, and 95.18 for group 1, 98.14, 98.95, 99.20, 97.72, 99.38, 98.69 for group 3, and 98.79, 99.23, 99.27, 98.95, 99,61, and 99.04 for group 5 (Table 2).

**Figure 6:**
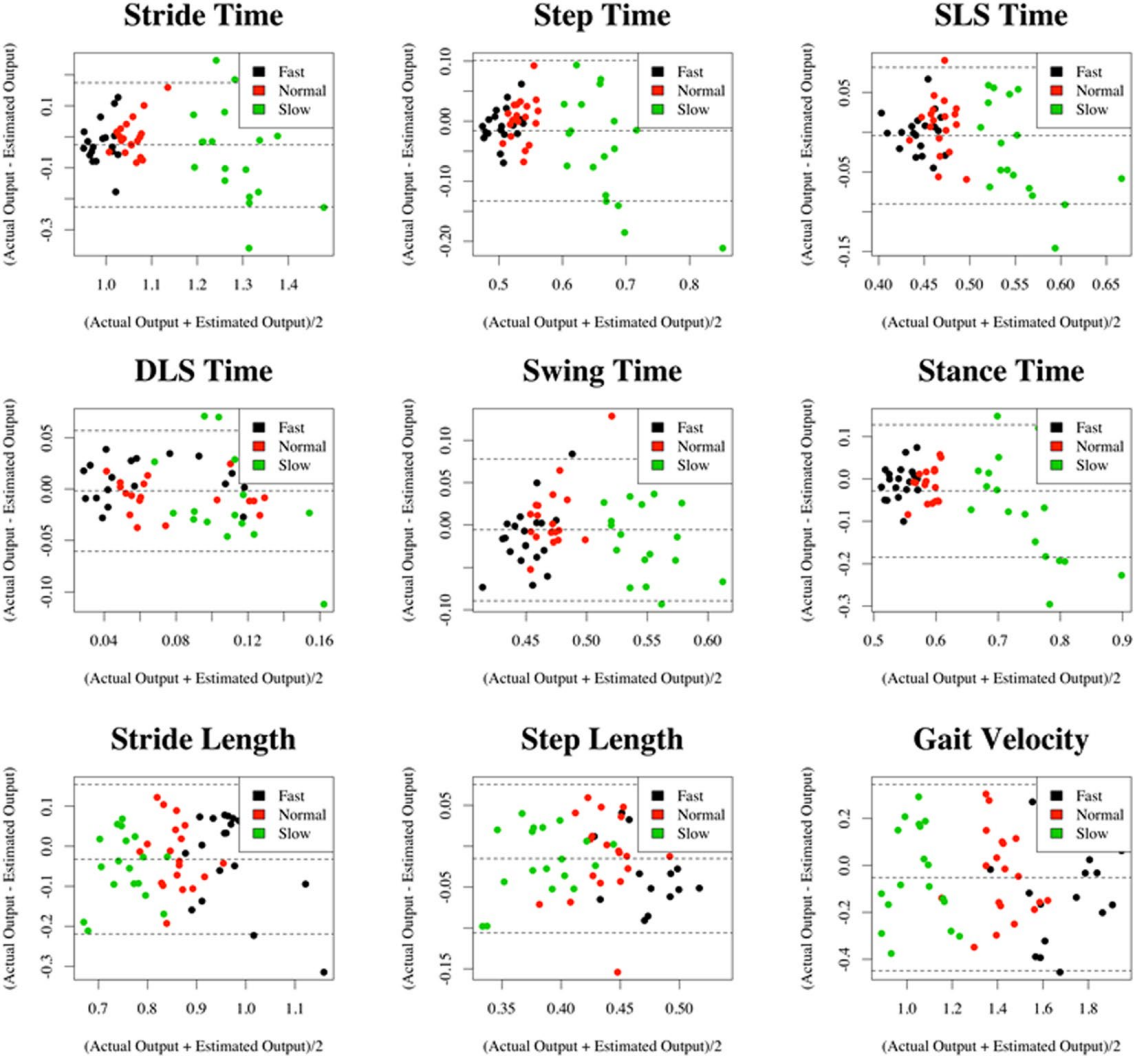
Bland-Altman plots for each of the output variables estimated by the DNN regressor.

The prediction errors of the gait spatial parameters showed much decrease when the inputs on the regressor increased. The MSEs and SDs of the stride and step length, and gait velocity were the highest in group 1; 0.019 ± 0.013, 0.008 ± 0.006, 0.133 ± 0.144. The MSEs were the lowest in group 5 showing 0.009 ± 0.006, 0.004 ± 0.002, and 0.05 ± 0.029. Those of group 3 were 0.010 ± 0.007, 0.004 ± 0.002, and 0.068 ± 0.037 (Table 1). The averaged accuracies of each spatial outputs were 97.805, 98.00, and 85.345 for group 1, 98.86, 99.19, 92.51 for group 3, and 99.001, 99.388, 94.815 for group 5 (Table 2). The detailed information on the actual gait temporo-spatial parameters and estimated outputs from the DNN regressor in group 5 were shown in Fig. 6 using Bland-Altman plots, which included the achieved mean accuracy and precision.

## Discussion

The study estimated the total of 27 gait outcomes including 9 temporo-spatial gait parameters at fast, normal, and slow walking speed, using foot characteristics including MLA and LLA along with other body anthropometric data.

The results deserve attention since they surpass the limits of the previous studies. For long, the curve of LLA had been neglected due to the absence of simultaneous measurement technique. The developed foot feature measurement system enables automatic analysis of the plantar surface of the foot by using a commercial RGB-D camera installed underneath the transparent scanning spot through the vision-based measurement approach in an efficient way^25^. The developed system also provides the foot anthropometric data such as foot length and width as well as morphological changes of the MLA and LLA curves simultaneously with 60 frames per second. It allows the quantitative assessment of the foot features such as foot length, height and arch angles in various conditions with a considerable accuracy and repeatability. The feasibility of the FFMS system was evaluated through our previous study^24,25^.

In this study, five sets of outputs were compared and an optimal input dataset was found in the proposed model. The foot characteristics were classified into 5 groups: (i) LLA features, (ii) MLA features, (iii) LLA + MLA features, (iv) group 3 + body anthropometric data, and (v) group 4 + labeling information indicating whether the subject is regular or athletes. The prediction accuracy of the DNN based regressor on gait temporal parameters was relatively poor in group 1 showing the averaged accuracy percentages from 93.44 to 97.42% (Table 2). The accuracies of group 2 were a lot higher than those of group 1 showing the accuracy range from 96.16 to 99.09%. In addition, the MSEs and SD of stride, step, SLS, and swing time in group 1 was about twice as high as group 2 (Fig. 5G and Table 1). This considerable increases in prediction accuracy that group 2 showed might be explained by the characteristics of their input dataset. Our previous study which investigated the correlation between the foot feature parameters and the gait temporo-spatial parameters confirmed that the MLA characteristics in OLS condition are highly correlated to the features of gait temporal parameters while those of LLA are related to gait spatial parameters^24^. The compelling contribution of MLA in gait temporal characteristics may have resulted in higher prediction accuracy in group 2 compared to group 1 under the same variable environment. Yet another error reduction found in group 3 can be explained by the independence of the MLA and LLA despite their proximity. Adopting both foot characteristics might have helped the regressor improve the prediction accuracy. The marginally improved accuracy found in group 4 and 5, which used both MLA and LLA as along with other body anthropometric data, supports this speculation of the importance of the foot characteristics in estimating the gait patterns.

For the most of the output variables, there was no considerable difference found between group 4 and 5 except for the DLS time. The averaged MSE of DLS time in group 5 was twice as low as the group 4. This might have been because of the mean DLS time of regular subjects being different from that of the semi-athlete subjects. The labeling information must have helped the DNN based regressor improve the prediction accuracy.

An up-to-date study^2^, which successfully demonstrated a CNN model translating an abstract information provided by IMU sensor mounted on a foot into context-related gait stride-parameters, proves that this novel method can outperform the currently available double integration approaches in estimating the stride, stance, and swing time. The root-MSEs (RMSE) and SDs of stride time, swing time, and stance time of the above mentioned study were 0.00 ± 0.07, 0.00 ± 0.05, and 0.00 ± 0.07, whereas the MSEs and SDs of our study at normal walking speed were 0.005 ± 0.003, 0.001 ± 0.001, and 0.002 ± 0.001. Although comparing these absolute values of the cited study with the results of our study is challenging since the former study only focused on two decimal places with different units, it is worth commenting that the SDs of our study were fairly smaller. It indicates that the approach we suggest can provide more consistent and precise estimation of the gait temporal aspects than the approach that the above-mentioned study suggested in^2^.

For the gait spatial parameters, the MSEs and SDs of stride length were relatively high in group 1 and 2 (0.019 ± 0.013 for group 1, and 0.014 ± 0.009 for group 2) compared to the other groups, and the group 4 showed the best performance on stride length prediction (0.007 ± 0.004). The prediction accuracies were from 97.80 to 99.01% (Table 2). The prediction of step length showed a similar pattern but the accuracy was slightly higher. When more variables were available as input dataset, the MSEs and SDs of the gait velocity decreased consequently. Nevertheless, the prediction accuracies of the gait velocity were relatively lower than those of stride and step length (from 85.35 to 94.82%). The more variables were input, the higher accuracy was achieved in estimating the gait velocity. It might have been due to the high variation of the gait velocity which may require more information in estimating.

When we compare our results on spatial parameters with those of the study mentioned above, the RMSE and SD of the stride length that the cited study showed was 0.15 ± 6.09 while our result at normal walking speed was 0.08 ± 0.05 when it was converted into RMSE for comparison^2^. From this comparison, we can conclude that our approach not only is more accurate and precise than the previous study but also takes various gait patterns at different walking speed into account. However, it is worth noting that there exists a discrepancy between^2^ and this study since former study uses a heterogeneous dataset of geriatric patients whereas the present study evaluates on healthy young and semi-athletes.

Table 2 shows the averaged accuracy of all the gait temporo-spatial parameters, group 3 seemed to be the most efficient and optimized input set for temporo-spatial gait parameter estimation despite the fact that group 3 had relatively smaller input variables of 18 compared to the 31 of group 4 and 33 of group 5. Thus, the regressor based on the group 3 was the most efficient in performing the estimation although it was relatively simple and less complex than other groups. Besides, the input variables applied to group 3 did not require manual measurement of the body anthropometric data and in turn, demanded less human labor. When the results of group 3 which had only the foot characteristics as variables were compared to those of group 4 and 5 which had other body anthropometric data in estimation, little difference was found. From this, we can conclude that foot characteristics serve as more dominant factors than another body anthropometric information in estimating personalized gait patterns.

Although this study successfully demonstrated that a new type of regressor model based on DNN can estimate temporo-spatial gait parameters quite effectively and accurately, the study bears a few inevitable limits. One is that the sample size was relatively small in both training and testing the network. Although there were 42 subjects participated in this study, there were only two maximum datasets of input and output per a subject. Of the 61 samples collected, 70% was used in training and the remaining 30% was used in testing. To address an overfitting issue caused by the sample size, the training and testing datasets were randomly shuffled and both processes were repeated for a hundred times. During the training process, a model which had shown the minimum errors was chosen as an optimal model and MSEs and SDs of this optimal model were calculated in the testing process.

As a study adopting DNN method, another limit that this study was not able to avoid was the use of black-box approach which provides little understanding of the generating mechanisms. Consequently, the results of this study are highly dependent on the training datasets^33,34^. The result can be understood in the current given data pool but it cannot be guaranteed that our DNN model can accurately provide the gait dynamics over time. As for the subjects, it should be commented that all recruited subjects were relatively young and healthy. The ones at old age or with muscular-skeletal injuries, foot-structural problems, or other neurological disorders were not included in this study. So estimating the gait parameters of the elderly or the injured was impossible. Further studies taking the old and injured into account would expand the analysis scope of gait and sports rehabilitation.

To conclude, this study developed a DNN based regressor that estimates gait temporo-spatial parameters using the foot structural features such as MLA and LLA measured in various movement conditions like sitting, standing, and OLS and the feasibility of the developed model was tested. The study not only proved that the accuracy of the developed regressor was comparable to those of the conventional approaches in practice but also assessed its feasibility. The cost-effectiveness and easiness of data collection can be the potential advantages of the developed model. By using this DNN based regressor, researchers can improve the data pool without using complex and expensive laboratory equipment. Further studies with various subject groups such as the old or patients with muscular-skeletal diseases or foot morphological disorders should be conducted to generalize the findings of this study.
